# Inhibition of Prostate Cancer DU-145 Cells Proliferation by *Anthopleura anjunae* Oligopeptide (YVPGP) via PI3K/AKT/mTOR Signaling Pathway

**DOI:** 10.3390/md16090325

**Published:** 2018-09-11

**Authors:** Xiaojuan Li, Yunping Tang, Fangmiao Yu, Yu Sun, Fangfang Huang, Yan Chen, Zuisu Yang, Guofang Ding

**Affiliations:** 1 Zhejiang Provincial Engineering Technology Research Center of Marine Biomedical Products, School of Food and Pharmacy, Zhejiang Ocean University, Zhoushan 316022, China; lxj19950329@163.com (X.L.); fmyu@zjou.edu.cn (F.Y.); gracegang@126.com (F.H.); cyancy@zjou.edu.cn (Y.C.); abc1967@126.com (Z.Y.); 2 Zhejiang Provincial Engineering Technology Research Center of Marine Biomedical Products, Zhejiang Ocean University Donghai Science and Technology College, Zhoushan 316000, China; suny@zjou.edu.cn; 3 Zhejiang Marine Fisheries Research Institution, Zhoushan 316021, China

**Keywords:** prostate cancer, *Anthopleura anjunae* oligopeptide, DU-145 cells, PI3K/AKT/mTOR signaling pathway

## Abstract

We investigated the antitumor mechanism of *Anthopleura anjunae* oligopeptide (AAP-H, YVPGP) in prostate cancer DU-145 cells in vitro and in vivo. Results indicated that AAP-H was nontoxic and exhibited antitumor activities. Cell cycle analysis indicated that AAP-H may arrest DU-145 cells in the S phase. The role of the phosphatidylinositol 3-kinase/protein kinase B/mammalian rapamycin target protein (PI3K/AKT/mTOR) signaling pathway in the antitumor mechanism of APP-H was investigated. Results showed that AAP-H treatment led to dose-dependent reduction in the levels of p-AKT (Ser473), p-PI3K (p85), and p-mTOR (Ser2448), whereas t-AKT and t-PI3K levels remained unaltered compared to the untreated DU-145 cells. Inhibition of PI3K/AKT/mTOR signaling pathway in the DU-145 cells by employing inhibitor LY294002 (10 μM) or rapamycin (20 nM) effectively attenuated AAP-H-induced phosphorylation of AKT and mTOR. At the same time, inhibitor addition further elevated AAP-H-induced cleaved-caspase-3 levels. Furthermore, the effect of AAP-H on tumor growth and the role of the PI3K/AKT/mTOR signaling pathway in nude mouse model were also investigated. Immunohistochemical analysis showed that activated AKT, PI3K, and mTOR levels were reduced in DU-145 xenografts. Western blotting showed that AAP-H treatment resulted in dose-dependent reduction in p-AKT (Ser473), p-PI3K (p85), and p-mTOR (Ser2448) levels, whereas t-AKT and t-PI3K levels remained unaltered. Similarly, Bcl-xL levels decreased, whereas that of Bax increased after AAP-H treatment. AAP-H also increased initiator (caspase 8 and 9) and executor caspase (caspase 3 and 7) levels. Therefore, the antitumor mechanism of APP-H on DU-145 cells may involve regulation of the PI3K/AKT/mTOR signaling pathway, which eventually promotes apoptosis via mitochondrial and death receptor pathways. Thus, the hydrophobic oligopeptide (YVPGP) can be developed as an adjuvant for the prevention or treatment of prostate cancer in the future.

## 1. Introduction

The ocean, with a total area of 360 million square kilometers, accounts for 70% of the earth’s surface area. The diversity of the marine environment is crucial for the unique metabolic pathways and genetic background of marine organisms, which produce active substances with special structures and functions [1,2]. Numerous studies have shown that marine organisms possess antithrombotic, antitumor, and antibacterial activities [3]. These unique marine bioactive substances have played significant roles in the development of innovative medicines.

Prostate cancer (PCa) is one of the most common malignancies of the male urinary system and is also the leading cause of cancer-related death in men [4]. In recent years, the incidence and mortality of PCa have increased in both western countries and in Asia [5]. Traditional surgical resection and chemotherapy are often accompanied by side effects, such as low survival rate, poor drug resistance, neurotoxicity, and hematological adverse events [6,7,8]. Recent studies have shown that bioactive peptides extracted from natural products usually possess low toxicity and anticancer activity [9]. Therefore, development of highly effective anti-prostate cancer peptides of low toxicity from natural products and elucidation of their anticancer mechanisms is urgently required.

Recently, the phosphatidylinositol 3-kinase/protein kinase B/mammalian rapamycin target protein (PI3K/AKT/mTOR) signaling pathway was shown to play a crucial role in malignant transformation and subsequent growth, proliferation, and metastasis of human tumors [10]. Numerous studies have shown that the PI3K signaling pathway is abnormally activated in a variety of cancers [11]. Inhibitors of this pathway are considered as potential drug candidates, and several of them are at different stages of clinical trials [12,13]. Chemical synthesis and extraction of PI3K pathway inhibitors from natural sources are two major methods of generating these inhibitors [14]. The main problems associated with chemical synthesis of inhibitors are their poor water solubility and absorption in the body and toxicity, which are circumvented by further chemically modifying these molecules [15]. Inhibitors from organisms are natural products with negligible toxicity and side effects, structural diversity, and multitarget activity [16,17]. Hence, new inhibitors, such as those targeting the PI3K/AKT/mTOR signaling pathway, are being extensively studied.

Anemones are rich in neurotoxic, cytotoxic, and cytolytic proteins and peptides. Toxic peptides from Sea anemone inhibit growth of various cancer cell lines, but studies on the activity of peptides extracted from sea anemone muscle are limited [18,19]. Previously, we showed that the *Anthopleura anjunae* oligopeptide (APP-H, YVPGP) exhibited anti-prostate cancer effect in vitro, the mechanism of which was also preliminarily investigated [20]. However, the antitumor mechanism of APP-H was not clearly understood. Hence, the antitumor mechanism of *Anthopleura anjunae* oligopeptide (AAP-H) on DU-145 cells, with emphasis on the PI3K/AKT/mTOR signal pathway, was further investigated in vitro and in vivo in this study.

## 2. Results and Discussion

### 2.1. Toxicity to Normal Cells

**Figure 1:**
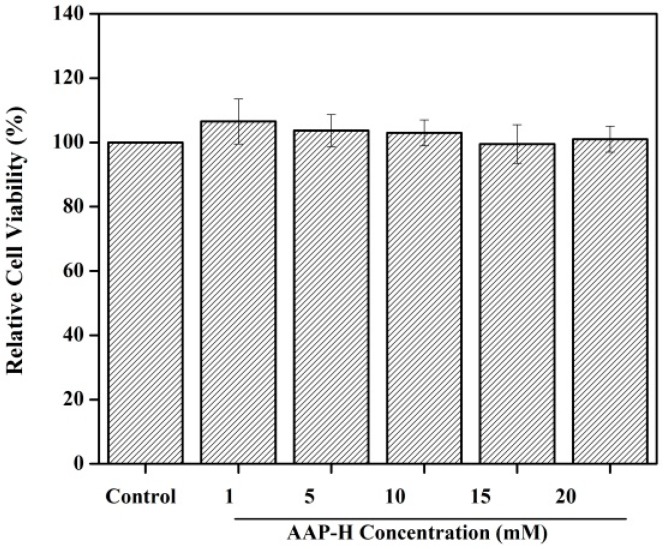
Toxicity of normal liver cells treated with *Anthopleura anjunae* oligopeptide (AAP-H). NIH-3T3 cells were treated with different concentrations of AAP-H (0, 1, 5, 10, 15, and 20 mM) for 24 h and the cell viability was assessed using the 3-(4,5-dimethyl-2 thiazolyl)-2,5-diphenyl-2*H*-tetrazolium bromide (MTT) assay. Each value represents the mean ± SD of three experiments.

Cell proliferation is the foundation of an organism’s growth, development, reproduction, and heredity. Perturbation in the balance between cell proliferation and apoptosis often leads to cancer [21]. Hence, inhibition of cell proliferation is an effective way of controlling cancer. In the previous study, we showed that AAP-H could inhibit the proliferation of prostate cancer DU-145 cells with a half-maximal inhibitory (IC_50_) concentration of 9.605 mM, 7.910 mM, and 2.298 mM at 24 h, 48 h, and 72 h, respectively [20]. However, the toxicity of AAP-H to normal cells was not studied. In this study, the fibroblast NIH-3T3 cells were used as the normal cells [22], which were added to culture solution containing different concentrations of AAP-H (0, 1, 5, 10, 15, and 20 mM) and incubated for 24 h. As shown in Figure 1, AAP-H showed no inhibitory effect on the fibroblast NIH-3T3 cells.

We observed that the rate of inhibition of DU-145 cells increased in a dose- and time-dependent manner [20], whereas the cell viability of NIH-3T3 cells was not affected under the same concentration. Thus, these observations indicated that AAP-H was nontoxic and exhibited antitumor activities. Huang et al. [23] reported that the inhibitory rate of a tripeptide (QPK) extracted from *Sepia ink* on DU-145 cells is 74.62% at 15 mg/mL. Song et al. [6] reported that the antiproliferative peptide (YALPAH) from *Setipinna taty* inhibited the DU-145 cells with an IC_50_ of 16.9 mM at 48 h. In comparison, the *Anthopleura anjunae* oligopeptides (YVPGP) showed lower IC_50_ values and better anticancer activity.

### 2.2. AAP-H Induced Cycle Arrest of DU-145 Cells

**Figure 2:**
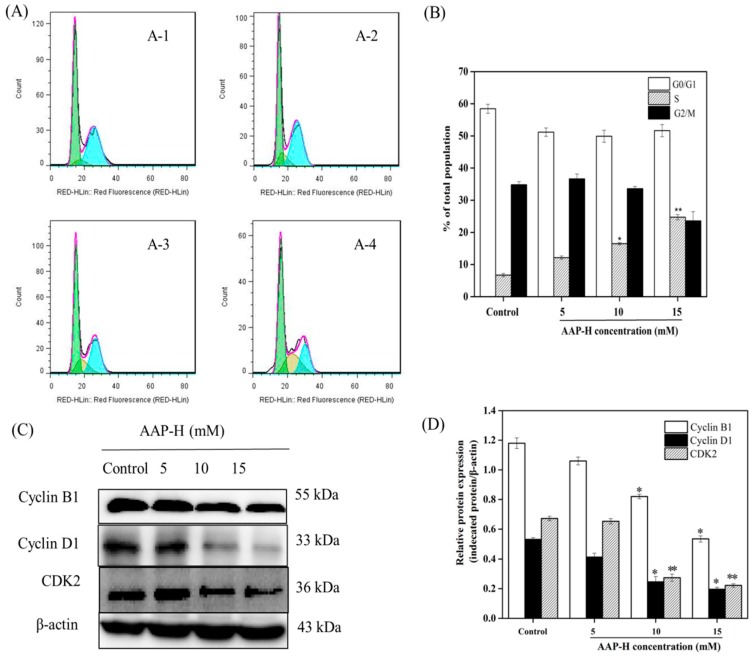
Flow cytometry of cell cycle phases (G0/G1, S and G2/M) distribution. (**A**) Percentage of DU-145 cells in S phase—A-1: control group, 7.3%; A-2: 5 mM AAP-H-treated group, 12.2%; A-3: 10 mM AAP-H-treated group, 17%, and A-4: 15 mM AAP-H-treated group, 20.9%. (**B**) The percentage of each phase in the cell cycle. Each experiment was repeated three times. (**C**) The levels of cyclin B1, cyclin D1, and CDK2 in DU-145 cells detected by western blotting analysis. (**D**) The expression levels of cyclin B1, cyclin D1, and CDK2 in each group analyzed by densitometry normalized to β-actin. * *p* < 0.05 and ** *p* < 0.001 vs. the control group.

The cell cycle is a tightly regulated and ordered event consisting of G1, S, G2, and M phases at the completion of which the newly replicated DNA is equally divided into daughter cells. The morphology and biochemistry of cells undergoing division change accordingly in these four stages [24,25]. The PI3K/AKT signaling pathways activate the cell-cycle-dependent protein kinase 2 (CDK-2) and CDK-4, which induces entry in S phase and DNA synthesis [26]. Evidence shows that AKT can actively and effectively regulate cell cycle progression from G2 to M phase and promote mitosis [27]. To investigate whether AAP-H affects cell cycle progression, DU-145 cells were treated with different concentrations of AAP-H (0, 5, 10, and 15 mM) for 24 h and assessed using flow cytometry. As shown in Figure 2, the number of cells in S phase increased in AAP-H treatment groups (*p* < 0.05) compared to the untreated controls. The percentages of cells in S phase after 24 h of AAP-H treatment (0, 5, 10, and 15 mM) were 7.3 ± 1.5%, 12.2 ± 1.4%, 17 ± 2.1%, and 20.9 ± 1.9%, respectively. To understand the mechanism underlying the AAP-H-induced S phase arrest, western blotting was used to analyze cell cycle regulatory proteins [28]. The levels of cyclin B1, cyclin D1, and CDK2 were dramatically reduced by varying degrees after treatment with high doses of APP-H (10–15 mM) (Figure 2).

Flow cytometric and western blotting analysis showed that AAP-H regulated the cell cycle by altering the distribution of DU-145 cells in different phases of the cycle. Compared to the blank control group, the proportion of G2/M phase cells decreased, whereas that of S phase cells increased. This indicated that AAP-H may block the progression of cancer cells from S to G2/M phase, which eventually leads to apoptosis.

### 2.3. AAP-H Suppressed the PI3K/AKT/mTOR Signaling Pathway in DU-145 Cells

Recent studies have indicated that PCa cells can be inhibited via the PI3K/AKT/mTOR signaling pathway. Meng et al. [29] reported that ursolic acid, a pentacyclic triterpenoid, can inhibit cell growth and induce apoptosis by regulating the PI3K/AKT/mTOR pathway in human PCa cells. Kumar et al. [30] demonstrated that rottlerin (rott), an active molecule isolated from Mallotus philippensis, inhibited autophagy and apoptosis in PCa stem cells. Yasumizu et al. [31] observed that hypoxic microenvironments promoted PI3K/Akt/mTOR signaling pathways and morphological changes in human castration-resistant prostate cancer (CRPC) cells via the PI3K/AKT/mTOR signaling pathway.

**Figure 3:**
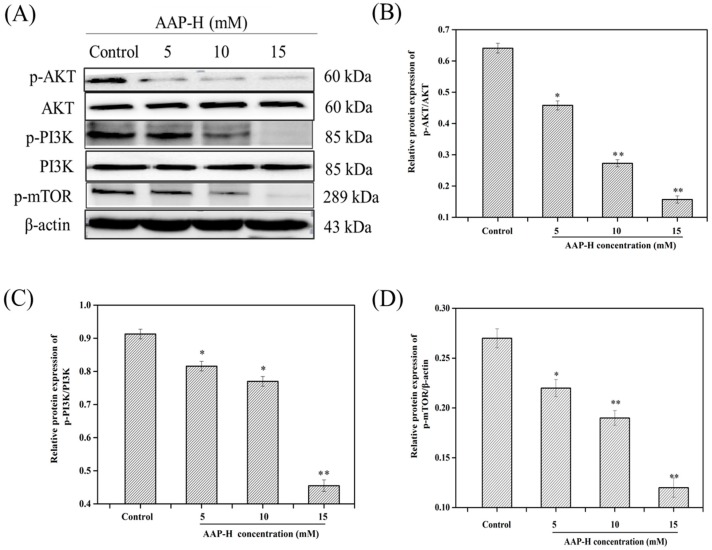
The changes of phosphatidylinositol 3-kinase/protein kinase B/mammalian rapamycin target protein (PI3K/AKT/mTOR) signaling pathway proteins—p-PI3K (p85), p-AKT (Ser473), p-mTOR (Ser2448), PI3K, AKT—in DU-145 cells after treating with AAP-H for 24 h. (**A**) The level of p-PI3K (p85), p-AKT (Ser473), p-mTOR (Ser2448) decreased in a dose-dependent way, and total PI3K and AKT were not changed compared to control groups. β-actin as a house-keeping protein was used for loading control. (**B**) Protein expression level of p-PI3K (p85) and PI3K that presented in AAP-H-treated DU-145 cells. (**C**) Protein expression level of p-AKT (Ser473) and AKT that presented in AAP-H-treated DU-145 cells. (**D**) Protein expression level of p-mTOR (Ser2448) that presented in AAP-H-treated DU-145 cells. * *p* < 0.05 and ** *p* < 0.001 vs. control.

Hence, the expression levels of p-PI3K (p85) [32], p-AKT (Ser473) [33], t-PI3K, t-AKT, and p-mTOR (Ser2448) [33] were investigated after 24 h treatment with AAP-H. As shown in Figure 3, AAP-H treatment led to a dose-dependent reduction in the levels of p-AKT (Ser473), p-PI3K (p85), and p-mTOR (Ser2448), whereas t-AKT and t-PI3K levels remained unaltered compared to the nontreated group. Multiple signaling pathways operate and cross-talk inside cells; hence, when one pathway is stimulated, the levels of proteins related to that pathway, as well as other associated pathways, change concomitantly [34].

### 2.4. PI3K/AKT/mTOR Signaling Involved in AAP-H-Induced Apoptosis

**Figure 4:**
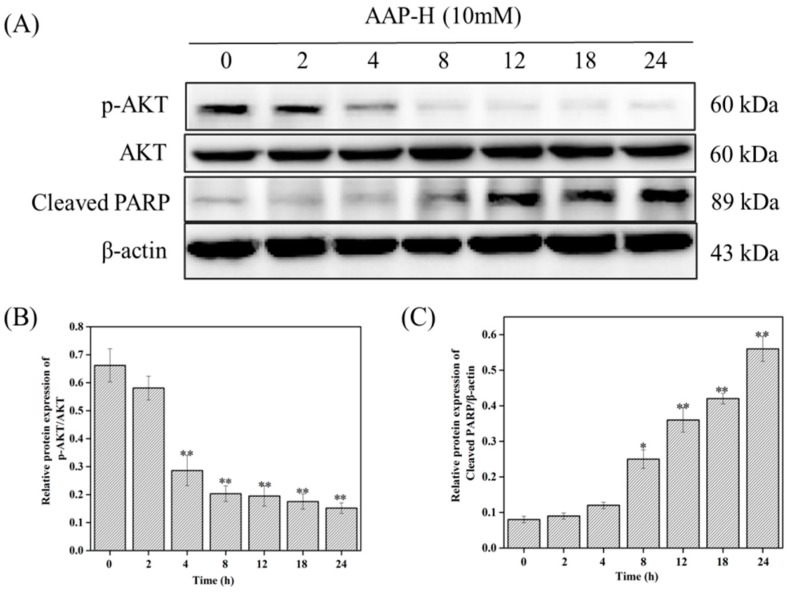
AAP-H affects PI3K/AKT/mTOR signaling pathway in different time points. (**A**) The protein expression level in the DU-145 cells treated with 10 mM AAP-H for 0, 2, 4, 8, 12, 18, and 24 h. (**B**) The expression levels of AKT/p-AKT (Ser473) analyzed using western blotting. (**C**) The expression levels of cleaved-PARP analyzed using western blotting. * *p* < 0.05 and ** *p* < 0.001 vs. control.

To further investigate whether AAP-H-induced apoptosis involves the PI3K/AKT/mTOR signaling pathway, the levels of p-AKT (Ser473), t-AKT, and cleaved-PARP were assessed using western blotting after DU-145 were treated with 10 mM AAP-H for 0, 2, 4, 8, 12, 18, and 24 h [35,36]. Results revealed that AAP-H reduced AKT phosphorylation in a time-dependent manner; however, AKT level remained unchanged. Cleaved-PARP level also increased in a time-dependent manner. Importantly, cleaved-PARP level started increasing concomitantly with decrease in AKT phosphorylation (Figure 4). These results indicated that PI3K/AKT/mTOR inhibition was an early event that was initiated prior to cell death induction.

**Figure 5:**
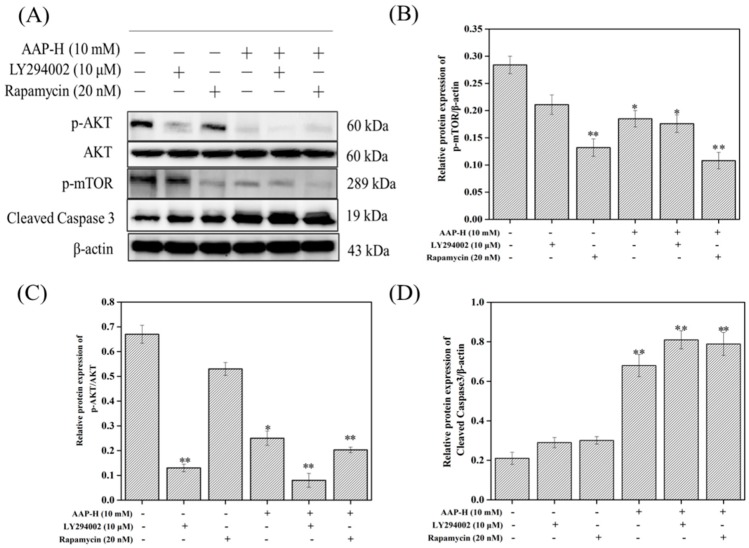
Effects of LY294002 or rapamycin on AAP-H-mediated alterations in PI3K/AKT/mTOR signaling and apoptosis-related protein. The DU-145 cells were preincubated with LY294002 (10 μM) or rapamycin (20 nM) for 2 h and then treated with 10 mM AAP-H for 24 h. (**A**) Western blot analysis of p-AKT, AKT, p-mTOR and Cleaved Caspase-3 levels. β-Actin as a house-keeping protein was used as the loading control. (**B**) The expression levels of p-mTOR analyzed using western blotting. (**C**) The expression levels of p-AKT/AKT analyzed using western blotting. (**D**) The expression levels of Cleaved Caspase-3 analyzed using western blotting. * *p* < 0.05 and ** *p* < 0.001 vs. control.

Furthermore, LY294002 [37] and rapamycin [38], were used to specifically inhibit the PI3K/AKT/mTOR signaling [39]. Figure 5 shows that the levels of phosphorylated AKT and mTOR were significantly reduced after treatment with LY294002 (10 μM) and rapamycin (20 nM), respectively. In addition, pretreatment with LY294002 and rapamycin effectively attenuated AAP-H-induced phosphorylation of AKT and mTOR. At the same time, inhibitor addition further elevated AAP-H-induced cleaved-caspase-3 levels. These results implied that the PI3K/AKT/mTOR signaling pathway is one of the potential mechanisms via which APP-H induces DU-145 cell apoptosis.

### 2.5. Effect of AAP-H on DU-145 Xenografts

**Figure 6:**
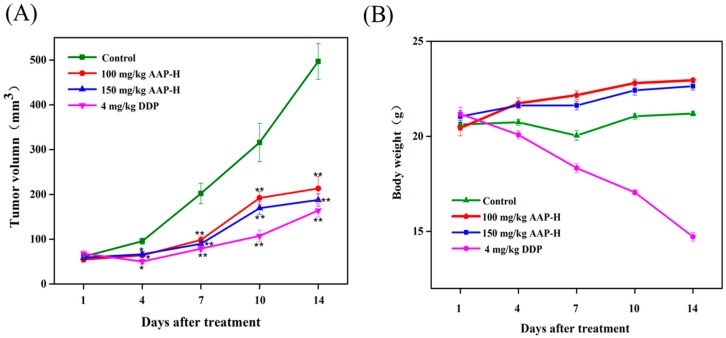
Inhibition of DU-145 prostate tumor growth by the APP-H. Compared to the control group, AAP-H exerted (**A**) antiproliferative effect on DU-145 cells in nude mice and (**B**) slightly increased mice body weight. Nude mice of control group, AAP-H group, and positive control group orally received saline, AAP-H (100 or 150 mg/kg/d), and DDP (4 mg/kg/d) for 14 days, respectively. * *p* < 0.05 and ** *p* < 0.001 vs. control.

To investigate the anticancer effects of AAP-H on DU-145 xenografts, physiological saline, AAP-H, and DDP were injected in nude mice for 14 days. Mice body weight and tumor volume were determined every three days. As shown in the Figure 6A, the growth rate of tumor volume in the AAP-H and DDP treatment groups was slower than that of the control group. The inhibition rate was 36.93 ± 3.9% in the AAP-H low dose group, 62.22 ± 6.2% in the AAP-H high dose group, and 66.96 ± 5.7% in the DDP group. Although DDP had the best inhibitory effect (Figure 6B), it elicited obvious toxic effects in nude mice. AAP-H not only reduced tumor weight but also slightly increased body weight and quality of life of nude mice during medication. Thus, we preliminarily concluded that AAP-H has no harmful effect on nude mice.

### 2.6. Immunocytochemistry

**Figure 7:**
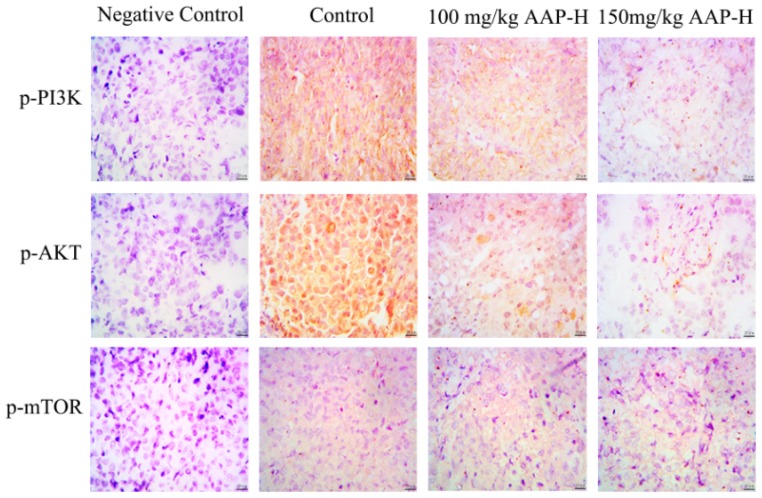
Expression of p-PI3K (p85), p-AKT (Ser473), and p-mTOR (Ser2448) in tumors was detected using immunocytochemistry. The primary antibody was replaced with phosphate buffered saline (PBS) in the negative control. DU-145 xenografts treated with non-AAP-H presented strong positivity, whereas those treated with 100 mg/kg AAP-H and 150 mg/kg AAP-H showed moderate and low positivity, respectively. Bar = 50 μm, 400×.

The expression of members of the PI3K/AKT/mTOR signaling pathway (p-AKT (Ser473), p-PI3K (p85) and p-mTOR (Ser2448)) in tumor tissue from nude mice was examined using immunocytochemistry assay [33]. As shown in Figure 7, p-AKT (Ser473), p-PI3K (p85), and p-mTOR (Ser2448) were detected in the cytoplasm and nucleus of the tumors; however, no expression was observed in the negative control group, whereas strong expression was observed in the positive control group. The positive expression started to decrease in the group treated with low dose of AAP-H (100 mg/kg). p-AKT (Ser473), p-PI3K (p85), and p-mTOR (Ser2448) levels were negligible and cell morphology was poor when the concentration of AAP-H was increased to 150 mg/kg. The results of immunohistochemistry were further confirmed by western blotting analysis.

Recent studies have shown that mutations in PI3KCA of PI3K play crucial roles in the formation of human tumors [40]. Phosphorylated PI3K triggers the production of the second messenger inositol triphosphate, further activating AKT, which can phosphorylate a variety of downstream factors such as enzymes, kinases, and transcription factors to promote tumor production [41]. AKT is an evolutionarily conserved serine/threonine kinase with three subtypes that are expressed to varying degrees in most human tissues. p-AKT is a typical pro-cancer factor, which can promote proliferation of tumor cells via p21, p27, and p53, inhibiting apoptosis, and promoting invasion, metastasis, and angiogenesis of tumor cells. Reports show that p-AKT is expressed in various malignancies, such as oral cancer, breast cancer, and non-small cell lung cancer [42,43,44]. mTOR is a direct substrate of AKT kinase, and serine 2480 is the site of phosphorylation of the latter on mTOR; the deletion of the residue in the C terminal regulatory region of the protein increases mTOR activity [41]. Hence, immunocytochemistry was used in this study to detect p-AKT in DU-145 cells. Our results showed that the expression of activated AKT, PI3K, and mTOR was increased in DU-145 xenografts, which resulted in continuous activation of the PI3K/AKT/mTOR pathway and rapid cell proliferation, thereby promoting tumor malignancy.

### 2.7. AAP-H Suppressed the PI3K/AKT/mTOR Signaling Pathway in DU-145 Xenografts

**Figure 8:**
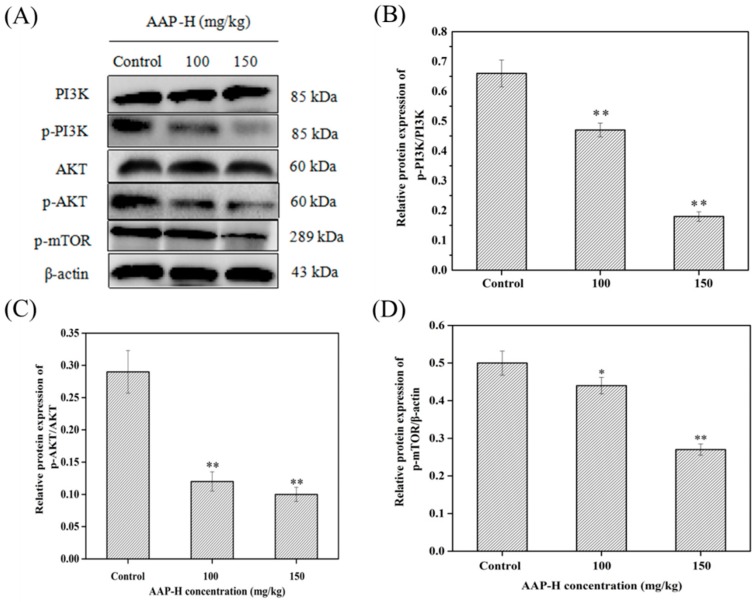
Changes in the levels of PI3K/AKT/mTOR signaling pathway proteins (p-PI3K (p85), p-AKT (Ser473), p-mTOR (Ser2448), PI3K, AKT) in DU-145 xenografts treated with AAP-H for 14 days. (**A**) The level of p-PI3K (p85), p-AKT (Ser473), p-mTOR (Ser2448) were reduced in a dose-dependent manner, whereas total PI3K and AKT were unaltered compared to control groups. β-actin as a house-keeping protein was used as the loading control. (**B**) p-PI3K (p85) and PI3K levels in AAP-H-treated DU-145 cells. (**C**) p-AKT (Ser473) and AKT levels in AAP-H-treated DU-145 cells. (**D**) p-mTOR (Ser2448) level in AAP-H-treated DU-145 cells. * *p* < 0.05 and ** *p* < 0.001 vs. control.

The PI3K/AKT/mTOR signaling pathway plays a crucial role in growth, proliferation, metastasis, and malignant transformation in human cancer [45]. It can induce tumor formation via the following mechanisms: (1) It inhibits autophagy [46]. For example, mTOR is a key regulator of the initiation stage of autophagy, and its activation inhibits autophagy [47]. (2) Activated AKT inhibits caspases 3 and 9 by phosphorylating their Ser196 and ultimately inhibiting apoptosis [48,49]. Furthermore, it inhibits the Bcl-2 family of proteins. Bad promotes apoptosis with Bcl-2 or Bcl-xL, and activated AKT is a highly effective Bad kinase that can be blocked by phosphorylation of Ser136 to induce apoptosis. (3) Promotion of tumor metastasis, cell movement, and angiogenesis are related to PI3K/AKT signal transduction. The above observations suggest that the PI3K/AKT/mTOR pathway is closely related to the caspase and Bcl-2 signaling pathways. Changes in the levels of key proteins of the PI3K/AKT/mTOR signaling pathway (p-PI3K (p85), p-AKT (Ser473), t-PI3K, t-AKT, and p-mTOR (Ser2448)) in DU-145 xenografts treated with AAP-H for 14 days were analyzed using western blotting. Compared to the control group, AAP-H treatment resulted in dose-dependent reduction in the levels of p-AKT (Ser473), p-PI3K (p85), and p-mTOR (Ser2448), whereas t-AKT and t-PI3K levels remained unaltered (Figure 8). These results were consistent with the in vitro results, indicating that AAP-H may inhibit the activity of DU-145 xenograft by regulating the PI3K/AKT/mTOR signal transduction pathway.

### 2.8. Western Blotting Analysis of Bcl-2 Family Members

**Figure 9:**
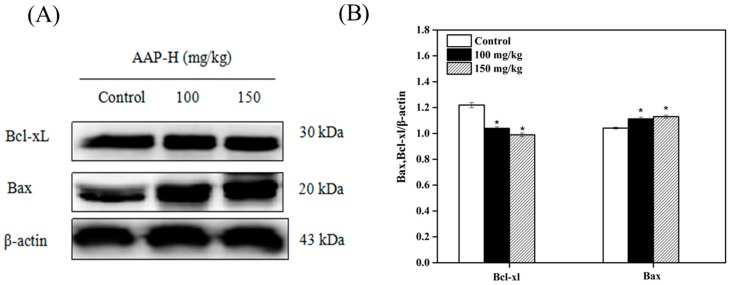
Western blotting showing the expression of Bcl-2 family members (Bcl-xL, Bax) in DU-145 xenografts treated with AAP-H for 14 days. (**A**) Western blotting analysis of Bcl-xL and Bax in DU-145 xenografts treated with AAP-H for 14 days. (**B**) The expression levels of Bcl-xL and Bax analyzed using western blotting. * *p* < 0.05 vs. control.

Apoptosis is triggered via the extrinsic (death receptor pathway) and intrinsic (mitochondrial pathway) pathways [50]. The Bcl-2 family of proteins, which regulates the release of cytochrome C from the mitochondria to the cytoplasm, is mainly composed of anti-apoptotic proteins (Bcl-2 and Bcl-xL) and apoptotic proteins (Bax, Bak). Anti-apoptotic and apoptotic proteins play opposite roles during the release of cytochrome C. For example, oligomeric Bax assists cytochrome C and other apoptotic proteins to pass through the mitochondrial membrane. However, Bcl-xL closes the voltage dependent anion channel (VDAC), which inhibits the release of cytochrome C from the Bax/VDAC channel [51]. To investigate whether apoptosis-related proteins participate in AAP-H-induced apoptosis, we analyzed the expression of Bcl-2 family members such as Bcl-xL and Bax. As shown in Figure 9, the level of the anti-apoptotic Bcl-xL was reduced, whereas that of the pro-apoptotic Bax was elevated post-AAP-H treatment. This indicated that AAP-H might affect apoptosis via the mitochondrial pathway.

### 2.9. Western Blotting of Caspases

The caspase family consists of cysteine-rich proteins, which are divided into initiator caspases (caspases 8 and 9) and executor caspases (caspases 3, 6, and 7). Caspases are activated by two major pathways. One involves the release of cytochrome C from the mitochondria to the cytoplasm, which eventually leads to apoptosis. The other pathway involves the death receptors [50].

**Figure 10:**
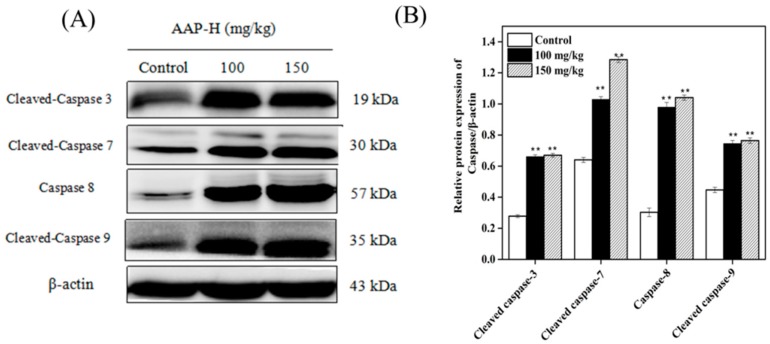
Western blotting showing the expression of Caspase family members (Caspase 3, 7, 8 and 9) in DU-145 xenografts after being treated with AAP-H for 14 days. (**A**) Western blotting analysis of Caspase 3, 7, 8 and 9 in DU-145 xenografts. (**B**) The expression levels of Caspase 3, 7, 8 and 9 analyzed using western blotting. ** *p* < 0.001 vs. control.

Death receptor is a transmembrane protein on the cell membrane, which is responsible for transferring extracellular stimulating signal to the corresponding ligand and inducing apoptosis [52]. Caspase 8, one of the initiators of apoptosis, plays a key role in mediating caspase activation in the death receptor pathway [53] by activating the downstream executors, caspases 3, 6, and 7. However, caspase 8 exists mainly in an inactive state in human cancers. Recently, caspase 8 expression has been analyzed from various perspectives [54,55]. Here, we investigated the levels of initiator and executor caspases in treated and untreated tumors. As shown in Figure 10, initiator caspase 8 and 9 were activated and expressed to similar extents in the presence of 150 mg/kg AAP-H. This was paralleled by increase in the expression of the downstream executor caspase 3 and 7. These results indicated that AAP-H promoted apoptosis of DU-145 cells via both the mitochondrial and death receptor pathways.

## 3. Materials and Methods

### 3.1. Materials and Reagents

The AAP-H (YVPGP) samples were purified from *Anthopleura anjunae* protein hydrolysates as mentioned previously [56] and stored at −20 °C until further use. The DU-145 cell lines and NIH-3T3 cell lines were purchased from the Cell Bank of Chinese Academy of Sciences. Powdered Ham’s F12/F-12 nutrient mixture (F12) medium was purchased from Gibco/BRL (Gaithersburg, MD, USA). Fetal bovine serum (FBS) was purchased from Sijiqing Biological Technology Co. (Hangzhou, China). 3-(4,5-dimethyl-2 thiazolyl)-2,5-diphenyl-2*H*-tetrazolium bromide (MTT) kits and cocktail 2 were purchased from Sigma Chemical Co. Ltd. (St. Louis, MO, USA). Antibodies against β-actin (cat. no. 4970S), caspase 3 (cat. no. CST9665T), caspase 7 (cat. no. CST9494S), caspase 8 (cat. no. CST4790T), caspase 9 (cat. no. CST9508T), AKT (cat. no. CST2920ST), p-AKT (Ser473) (cat. no. CST4060T), PI3K (cat. no. CST4257T), p-mTOR (Ser2448) (cat. no. CST5536T), p-PI3K (p85) (cat. no. CST4228T), Bax (cat. no. CST5023T), Bcl-xL (cat. no. CST2764T), PARP (cat. no. CST9532T), CDK2 (cat. no. CST2546T), cyclin D1 (cat. no. CST2978T) and cyclin B1 (cat. no. CST12231T) were purchased from Cell Signaling Technology (Boston, MA, USA). Cell cycle detection kits were purchased from BestBio Biological Technology Co. (Shanghai, China). All other reagents used in the present study were of analytical grade.

### 3.2. Cell Toxicity Assay

The effect of AAP-H on the toxicity of NIH-3T3 cell viability was detected using the MTT assay according to the method described by Tang et al. [57], with slight modifications. In brief, the NIH-3T3 cells were first seeded in a 96-well (5 × 105 cell/mL) plate (Costar Corning, Rochester, NY, USA). After incubation for 24 h, 200 μL fresh culture medium containing different final concentrations of AAP-H (0, 1, 5, 10, 15, and 20 mM) was added to each well. After 24 h incubation, the cells were treated with 200 μL MTT reagents and incubated for another 4 h. Finally, the formazan crystals were dissolved in 100 μL dimethyl sulfoxide (DMSO), and the absorbance at 490 nm was recorded using an enzyme-linked immunosorbent assay (ELISA) reader (SpectraMa, Molecular Devices Co., San Jose, CA, USA). Different final concentrations of AAP-H (0, 1, 5, 10, 15, and 20 mM) were used for treatment prior to the MTT assay.

### 3.3. Determination of Cell Cycle Arrest

To investigate the cytotoxic effect of AAP-H on DU-145 cells, the relative sensitivities of the different phases of the cell cycle were observed using the cell cycle arrest test described by Chen et al. [58], with slight modifications. First, the cells were placed in a 6-well plate and different final concentrations (0, 5, 10, and 15 mM) of APP-H were added when the cells had adhered to the bottom of the plate. After 24 h incubation, the cells were collected by centrifugation, RNase A was added to the cell pellet, and incubated in a water bath at 37 °C for 30 min. Three hundred and fifty microliters of propidium iodide was added to the tubes in dark and incubated at 4 °C for another 40 min. Finally, the cells were sorted using a flow cytometer (Becton Dickinson, NJ, USA).

### 3.4. Western Blot Analysis

DU-145 cells were treated with culture solution containing different concentrations of AAP-H (0, 5, 10, and 15 mM). Then, the treated cells were incubated in a CO_2_ incubator for 24 h. After washing thrice with phosphate buffered saline (PBS) (the tumor tissues were placed in a mortar, liquid nitrogen was added, and the tumor was rapidly ground and collected in a centrifuge tube), 200 μL radioimmunoprecipitation assay (RIPA) lysis solution containing 1% phosphatase inhibitor and phenylmethane sulfonyl fluoride (PMSF) was added to each group. The cracking liquid was transferred into a 1.5 mL centrifuge tube and sonicated for 30 min. Subsequently, the cell lysates were collected by centrifugation at 4 °C (12,000 rpm, 15 min). The total protein content of the supernatant was measured using the bicinchoninic acid (BCA) kit. The samples were then denatured by heating in a boiling water bath for 10 min, and the total protein (50 μg) was separated on preprepared 12% sodium dodecyl sulfate-polyacrylamide gels, followed by transfer to polyvinylidene fluoride membrane (Millipore, Billerica, MA, USA). The membranes were stained with Ponceau S and then blocked with 5% nonfat milk for 1 h. After that, the membranes were incubated with the 1:1000 diluted primary antibodies against caspases 3, 7, 8, and 9, AKT, p-AKT (Ser473), PI3K, p-PI3K (p85), p-mTOR (Ser2448), Bax, Bcl-xL, PARP, CDK2, cyclin B1, and cyclin D1 in 5% *w*/*v* nonfat milk at 4 °C with gentle shaking for 16 h. Finally, the 1:1000 diluted secondary antibodies (goat anti-rabbit and goat anti-mouse) were added and incubated at room temperature for 1 h. The target bands were detected using enhanced chemiluminescence and quantified by densitometry as shown in DRAFT-alpha view using the Image J 1.38 software (NIH, Bethesda, MD, USA). β-actin was used as the loading control.

### 3.5. Effect of AAP-H on DU-145 Xenografts

Male BALB/c nude mice (nu/nu) were obtained from the Zhejiang Laboratory Animal Center (Hangzhou, China) and housed in specific pathogen-free conditions. DU-145 cells were cultured in Ham F12 medium (10% FBS) at 37 °C in a 5% CO_2_ incubator. A suspension of DU-145 cells (in the logarithmic growth stage) were then prepared (0.2 mL, with 1 × 10^6^ cells) and injected into the right armpit of nude mice in sterile environment. The tumor nodes appeared after 7 days, which confirmed the successful establishment of the DU-145 tumor-bearing nude mouse model. The body weight was measured after every three days, and the tumor volume was calculated according to the following formula: volume (mm^3^) = 1/6 × π × width^2^ × length. When the average tumor volume reached 60 mm^3^, the mice were randomly divided into four groups with five mice in each group (negative control, positive control treated with 4 mg/kg cisplatin (DDP), and two treatment groups with 100 mg/kg and 150 mg/kg AAP-H). All mice were injected intraperitoneally for 14 days. The experiments were performed according to the guidelines of the Institutional Animal Care and Use Committee of the Zhejiang Ocean University (SYXK, 2014-0013) and adhered to the code of the World Medical Association (Declaration of Helsinki).

### 3.6. Immunocytochemistry (IHC)

The animal experiments were divided into four groups, and each group contained five nude mice. All animals were euthanized, and the three transplanted tumors were selected randomly and cut into 1-cm^3^ pieces for IHC. IHC was performed as follows: First, the tumors were placed in 4% paraformaldehyde for 24 h and washed with flowing water overnight. Second, the tumors were dehydrated using an ethanol gradient (75%, 95%, and 100%) for 5 min and transparentized with xylene for 40 min. Then, the tumors were put in paraffin to maintain constant temperature (60 °C) for 1 h. Finally, the tumors were put into a square box, fluid wax was poured into these boxes, and the boxes were refrigerated at 4 °C. Subsequently, 4-μm slices were prepared using a Leica slicer (RM2135, Leica Instruments GmbH, Wetzlar, Germany). The adhered slices were peeled off using a spreading machine (III1210, Leica Instruments GmbH, Wetzlar, Germany) and processed using a baking machine (II1220, Leica Instruments GmbH, Wetzlar, Germany). The paraffin section was used for IHC.

IHC for determining the expression of p-PI3K (p85), p-AKT (Ser473), and p-mTOR (Ser2448) was performed according to Luo et al. [59], with minor modifications. Briefly, 4-μm thick sections were dewaxed with xylene for 15 min. Antigen repair of tumor sections was performed using EDTA (pH 8.0) buffer under high temperature and pressure for 3 min. The endogenous biotin in the tumor sections were blocked according to the instructions of the IHC biotin block kit (Maxin Co., Hong Kong, China). Then, the tumor sections were incubated with prediluted primary antibodies against p-PI3K (p85) (1:50), p-AKT (Ser473) (1:50), and p-mTOR (Ser2448) (1:50) in a mixture containing 3% bovine serum albumin (BSA) (*w*/*v*), 1× TBS and 0.1% Tween 20 at 4 °C for 16 h. Horse radish peroxidase (HRP)-labeled goat anti-rabbit monoclonal secondary IgG (H + L) against p-PI3K (p85) (1:1000), p-AKT (Ser473) (1:1000), and p-mTOR (Ser2448) (1:1000) were used, and the signal was detected using the DAB chromogenic kit (Maxin Co., Hong Kong, China). Sections were re-stained with hematoxylin, dehydrated, transparentized with xylene, and sealed with a resin. The primary antibodies were replaced with PBS in the negative control. Antigen expression was analyzed using light microscopy (Olympus, Tokyo, Japan).

## 4. Conclusions

**Figure 11:**
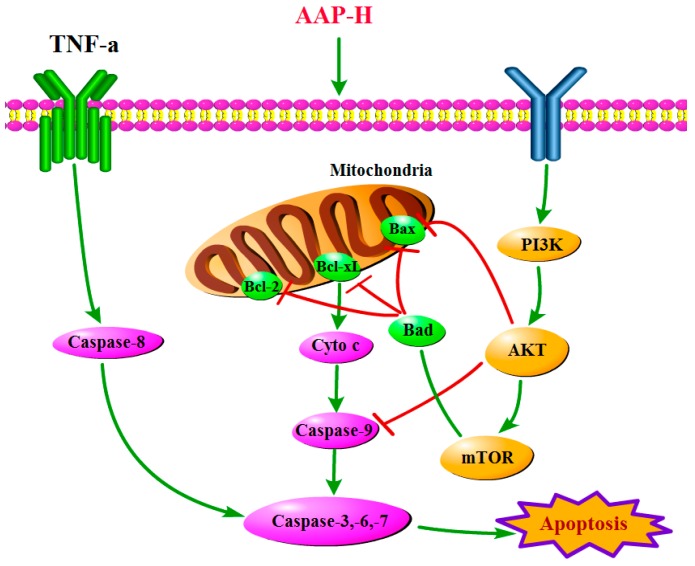
AAP-H inhibits DU-145 cell proliferation via the PI3K/AKT/mTOR signaling pathway.

AAP-H exhibited anticancer activity on DU-145 prostate cancer cells by targeting the PI3K/AKT/mTOR signaling pathway and was not toxic for normal fibroblast cells. In addition, AAP-H showed good antiproliferative effect on DU-145 cells. The antitumor mechanism of APP-H on DU-145 cells may involve regulation of the activity of the PI3K/AKT/mTOR signaling pathway, which eventually promotes apoptosis via both the mitochondrial and death receptor pathways (Figure 11). Therefore, the hydrophobic oligopeptide (YVPGP) will be further studied for use as a functional food or adjuvant for prevention or treatment of prostate cancer.
